# Antimicrobial, Antioxidant, and Cytotoxic Properties of Vasicine Acetate Synthesized from Vasicine Isolated from *Adhatoda vasica* L.

**DOI:** 10.1155/2015/727304

**Published:** 2015-01-14

**Authors:** V. Duraipandiyan, N. A. Al-Dhabi, C. Balachandran, S. Ignacimuthu, C. Sankar, K. Balakrishna

**Affiliations:** ^1^Department of Botany and Microbiology, Addiriyah Chair for Environmental Studies, College of Science, King Saud University, P.O. Box 2455, Riyadh 11451, Saudi Arabia; ^2^Division of Microbiology, Entomology Research Institute, Loyola College, Chennai 600034, India; ^3^Visiting Professor Programme, Deanship of Scientific Research, College of Science, King Saud University, Saudi Arabia

## Abstract

*Adhatoda vasica* (L.) (Acanthaceae) is used in the indigenous system of medicine in India. The alkaloid Vasicine was isolated from ethanolic extract of the leaves of *A. vasica* using column chromatography. Vasicine acetate was obtained by acetylation of Vasicine. Vasicine acetate exhibited good zone of inhibition against bacteria: 10 mm against *E. aerogenes*, 10 mm against *S. epidermidis*, and 10 mm against *P. aeruginosa*. Vasicine acetate showed minimum inhibitory concentration values against bacteria: *M. luteus* (125 *μ*g/mL), *E. aerogenes* (125 *μ*g/mL), *S. epidermidis* (125 *μ*g/mL), and *P. aeruginosa* (125 *μ*g/mL). The radical scavenging activity of Vasicine acetate was the maximum at 1000 *μ*g/mL (66.15%). The compound showed prominent cytotoxic activity in vitro against A549 lung adenocarcinoma cancer cell line. Quantification of Vasicine and Vasicine acetate by HPLC-DAD analysis showed their contents to be 0.2293% and 0.0156%, respectively, on dry weight basis of the leaves. Vasicine acetate could be probed further in drug discovery programme.

## 1. Introduction

Natural products still remain the most important source for discovery of new and potential drug molecules. Medicinal plants are important sources of practical drugs for people throughout the year. Nature acts as a prominent reservoir for new and novel therapeutics. The emergence of drug-resistant pathogens and the increase in diseases affecting the immune system have greatly intensified the need to investigate new bioactive metabolites for potential pharmaceutical and industrial applications [1, 2]. Every year, at least 200,000 people die worldwide from cancer related to their workplace [3].

*Adhatoda vasica* (L.) Nees (Acanthaceae), known commonly as Malabar nut tree, is a shrub growing throughout the Indian peninsula. The plant is used in the indigenous system of medicine in India and is a well-known expectorant in both Ayurvedic and Unani Systems of Medicine [4, 5]. The leaves are used to treat malarial fever, chronic fever, intrinsic hemorrhage, cough, asthma, leprosy, skin diseases, and piles [6]. The plant is reported to show abortifacient [7], antimicrobial [8, 9], and antitussive activities [10]. The crude extract of* A. vasica* leaves was found to have conspicuous antifeedant and toxic effects on the larvae of* S. littoralis* [11]. The plant contains alkaloids such as Vasicine, vasicinone, deoxyvasicine, vasicol, adhatodinine, and vasicinol [12]. Other constituents include vitamin C, saponins, flavonoids as well as steroids, and fatty acids [13]. Vasicine is reported to have bronchodilatory, respiratory stimulant, and uterine stimulant effects [14]. Vasicine acetate showed antimycobacterial activity [15]. Essential oils of the leaves of* A. vasica* are also known to contain ketone, terpene, and phenolic ether which have antitumor, antioxidant, antiaging, antimutation, and sedative effects; the high phenolic content of essential oils contributes to their antimicrobial properties [13]. In the present communication we report the antimicrobial, antioxidant, and anticancer effects of Vasicine acetate acetylated from Vasicine obtained from* A. vasica* leaves.

## 2. Materials and Methods

### 2.1. Collection of the Plant Material

The plant material was collected from Vandalur, Chennai, Tamil Nadu, during the month of June 2010. The plant was identified by Dr. V. Duraipandiyan. A voucher specimen (No. ERI/ETHPH/TA/171) was deposited at the herbarium of the institute.

#### 2.1.1. Extraction

Shade dried and coarsely powdered leaves of* A. vasica* (2 kg) were extracted with ethanol, twice in the cold by cold percolation method (48 h). The extract was filtered through Whatman No. 1 filter paper and distilled on a water bath. It was concentrated to a dark green residue which was finally dried in vacuum (yield 100 g).

#### 2.1.2. Column Chromatography

A portion of the above extract (100 g) was chromatographed over silica gel (Acme's, 100–200 mesh) packed in chloroform. The column was eluted with chloroform and chloroform: methanol mixtures with increasing amounts of methanol. Further elution of the column with chloroform : methanol 4 : 1 gave Vasicine which crystallised from methanol as colorless solid (m.p 210, Lit.m.p 212-213) (98% pure). It gave a single spot on a TLC over silica gel (RF-0.48) with chloroform : methanol 3 : 1 developing system (yield 2.5 g). The spot turned orange red on spraying with Dragendorff's reagent.

### 2.2. Acetylation of Vasicine to Vasicine Acetate

Vasicine (1 g) was dissolved in 25 mL of acetic anhydride and kept in the fridge overnight. Five drops of concentrated H_2_SO_4_ were added to the ice cold solution, stirred well, and heated on a water bath (50°C) for 30 min. It was then poured into crushed ice, diluted with water (250 mL), and extracted with chloroform (2 × 250 mL) in a separating funnel. The combined chloroform extract was washed with water, dipped, and dried over anhydrous sodium sulphate. It was then distilled on a water bath and the residue was washed with little n-hexane. Then the residue was crystallised from chloroform to get Vasicine acetate as colorless crystals (m.p 120, Lit.m.p 122). It gave a single spot on TLC (RF-0.6) with n-hexane : ethyl acetate 2 : 1 as the developing system. The spot turned orange red on spraying with Dragendorff's reagent.

### 2.3. Preparation of Sample for HPLC Analysis

Sample (ethanol extract) (92.4 mg in 5 mL) and standard Vasicine and Vasicine acetate (3.3 mg in 10 mL) were dissolved in methanol. The solutions were filtered through a membrane filter (pore size 0.20 *μ*m) prior to HPLC analysis.

#### 2.3.1. Quantification of Vasicine and Vasicine Acetate by HPLC-DAD Analysis

HPLC analysis was carried out on a Waters Alliance 2695 separations module with photodiode array detector (Waters, 2996). The LC column was an YMC pack ODS A (150 mm × 4.6 mm, 3.5 *μ*m). Two mobile phases A and B were used at flow rate of 1.0 mL/min. The mobile phase was filtered through a 0.45 *μ*m filter and degassed by vacuum, followed by sonication. Mobile phase A consisted of water with 0.1% orthophosphoric acid and mobile phase B was acetonitrile. Separation was carried out at room temperature. A gradient was used, starting at 95% A for 5 min, changing to 10% A linearly in 10 min. After elution the column was reequilibrated for 5 min under the initial conditions. The HPLC profile of* A. vasica* ethanol extract was compared with that of standard compound, Vasicine acetate, at a specific wavelength where the standards were best detected, that is, 270 nm and also by comparing with the UV spectra, extracted from the PDA detection, of the standards with the corresponding spectra of the respective peaks of the* A. vasica* ethanol extract. Vasicine acetate were run at six concentrations (10 *μ*g–500 *μ*g/mL) and found to be linear in the range with a correlation coefficient (*R* ^2^) of 0.99961. The estimation of Vasicine acetate content in the extract was performed using linear regression analysis. Similarly Vasicine was estimated, the detector wavelength being 280 nm. The standard solution was prepared by dissolving 2 mg of the compound in 100 mL methanol.

### 2.4. Instruments

All melting point values are uncorrected and taken by open capillary method on a heating block Instrument. UV-Visible spectra were taken in methanol on Thermo Fisher instrument. IR FT-IR spectra were taken on a PerkinElmer grating spectrometer in KBr disc. ^1^H and ^13^C NMR were taken in CDCL_3_ on a Bruker Instrument at 400 and 100 MHz, respectively. The chemicals are given in delta scale with TMS as an internal standard.

### 2.5. Microbial Organisms

The following Gram negative, Gram positive bacteria, clinical isolates, and fungi were used for the experiment: Gram negative bacteria are* Shigella flexneri* MTCC 1457,* Salmonella paratyphi-B, Klebsiella pneumoniae* MTCC 109,* Pseudomonas aeruginosa* MTCC 741,* Proteus vulgaris* MTCC 1771, and* Salmonella typhimurium* MTCC 1251; Gram positive bacteria are* Bacillus subtilis* MTCC 441,* Micrococcus luteus* MTCC 106,* Enterobacter aerogenes* MTCC 111,* Staphylococcus aureus* MTCC 96, and* Staphylococcus epidermidis* MTCC 3615; clinical isolates are* Escherichia coli* (ESBL-3984, Extended Spectrum Beta Lactamase),* Escherichia coli* (ESBL-3904),* Klebsiella pneumoniae* (ESBL-3971),* Klebsiella pneumoniae* (ESBL-75799),* Klebsiella pneumoniae* (ESBL-3894),* Klebsiella pneumoniae* (ESBL-3967), and* Staphylococcus aureus* (MRSA-methicillin resistant). The reference cultures were obtained from the Institute of Microbial Technology (IMTECH), Chandigarh, India; fungi are* Candida albicans* MTCC 227,* Malassezia pachydermatis, Trichophyton mentagrophytes* 66/01,* Scopulariopsis *sp. 101/01,* Trichophyton rubrum* 57/01,* Aspergillus flavus,* and* Botrytis cinerea*. All the cultures were obtained from the Department of Microbiology, Christian Medical College, Vellore, Tamil Nadu, India. Bacterial inoculums were prepared by growing cells in Mueller Hinton broth (MHB) (Himedia) for 24 h at 37°C. The filamentous fungi were grown on sabouraud dextrose agar (SDA) slants at 28°C for 10 days and the spores were collected using sterile double distilled water and homogenized. Yeast was grown on sabouraud dextrose broth (SDB) at 28°C for 48 h.

#### 2.5.1. Antimicrobial Activity

Antibacterial and antifungal activities were carried out using disc diffusion method [16]. Petri plates were prepared with 20 mL of sterile Mueller Hinton agar (MHA) (Himedia, Mumbai). The test cultures were swabbed on the top of the solidified media and allowed to dry for 10 min and a specific amount of the compound was added to each disc. The loaded discs were placed on the surface of the medium and left for 30 min at room temperature for compound diffusion. Negative control was prepared using respective solvents. Streptomycin was used as positive control for bacteria and Ketoconazole as positive control for fungi. The plates were incubated for 24 h at 37°C for bacteria and for 48 h at 28°C for fungi. Zones of inhibition were recorded in millimetres and the experiment was repeated twice.

#### 2.5.2. Minimum Inhibitory Concentration (MIC)

Minimum inhibitory concentration studies of the compounds were performed according to the standard reference methods for bacteria [17] and filamentous fungi [18]. The required concentrations (500, 250, 125, 62.5, 31.25, 15.62, and 7.81 *μ*g/mL) of the compound were dissolved in DMSO (2%) and diluted to give serial twofold dilutions that were added to each medium in 96 well plates. An inoculum of 100 *μ*L from each well was inoculated. The antifungal agent Ketoconazole for fungi and antibacterial agent Streptomycin for bacteria were included in the assays as positive controls. For fungi, the plates were incubated for 48 to 72 hours at 28°C and for bacteria the plates were incubated for 24 h at 37°C. The MIC for fungi was defined as the lowest extract concentration, showing no visible fungal growth after incubation time. 5 *μ*L of tested broth was placed on the sterile MHA plates for bacteria and incubated at respective temperature. The MIC for bacteria was determined as the lowest concentration of the compound inhibiting the visual growth of the test cultures on the agar plate.

#### 2.5.3. DPPH Radical Scavenging Assay

DPPH (2,2-diphenyl-1-picrylhydrazyl) radical scavenging activity of Vasicine acetate was determined based on the method of Wang et al. [19]. 40 *μ*L of various concentrations (125–1000 *μ*g/mL) of Vasicine acetate was added to ethanolic solution of DPPH (0.1 M, 2960 *μ*L). The absorbance of reaction mixture was measured at 517 nm after 30 minutes of incubation in the dark at room temperature. The free radical scavenging activity was calculated as follows:

(1) $$\begin{array}{c} {\text{DPPH}}^{∙}\;\text{scavenging}\;\text{activity}=\left[\left(A_{C}-\frac{A_{S}}{A_{C}}\right)\times 100\right], \end{array}$$

where *A*_*C*_ is the absorbance of the control and *A*_*S*_ is the absorbance of the extract/standard (BHT). The experiment was run in triplicate and the result was reported as mean ± standard deviation.

#### 2.5.4. Cytotoxic Properties of Vasicine Acetate

A549 human adenocarcinoma cancer cell line was obtained from National Institute of Cell Sciences, Pune. A549 lung adenocarcinoma cancer cell line was maintained in complete tissue culture medium DMEM with 10% fetal bovine serum and 2 mM L-glutamine, along with antibiotics (about 100 IU/mL of penicillin, 100 *μ*g/mL of Streptomycin) with the pH adjusted to 7.2. The cytotoxicity was determined according to the method of Balachandran et al. [20] with some changes. Cells (5 × 10^3^/well) were seeded in 96 well plates containing medium with different concentrations such as 2000, 1000, 500, 250, 125, and 62.5 *μ*g/mL. The cells were cultivated at 37°C with 5% CO_2_ and 95% air in 100% relative humidity. After various durations of cultivation, the solution in the medium was removed. An aliquot of 100 *μ*L of medium containing 1 mg/mL of 3-(4,5-dimethylthiazol-2-yl)-2, 5-diphenyl-tetrazolium bromide (MTT) was loaded to the plate. The cells were cultured for 4 h and then the solution in the medium was removed. An aliquot of 100 *μ*L of DMSO was added to the plate, which was shaken until the crystals were dissolved. The cytotoxicity against cancer cells was determined by measuring the absorbance of the converted dye at 540 nm in an ELISA reader. Cytotoxicity of each sample was expressed as IC_50_ value. The IC_50_ value is the concentration of test sample that causes 50% inhibition of cell growth, averaged from three replicate experiments.

### 2.6. Statistical Analysis

DPPH and cytotoxic properties of isolated compounds were statistically analyzed by Duncan multiple range test at *P* = 0.05 with the help of SPSS 11.5 version software package.

## 3. Results and Discussion

The ethanol extract of* A. vasica* leaves was subjected to column chromatography. The column was eluted with chloroform and chloroform : methanol mixtures with increasing amounts of methanol. The fraction was further purified and the compound Vasicine was isolated. The structure was elucidated using spectroscopic methods:* M/z* 188. IR: *υ*_max⁡_KBr CM^−1^: 3064 (hydroxyl), 2847, 1631 (>C=N), 1465, 1303, 1183, 1110, 871, 760. ^1^H NMR (*δ*, CDCL_3_, 400 MHz): 3.21 (1H, m, H-1*α*), 3.40 (1H, m, H-1*β*), 2.35 (1H, m, H-2*α*), 2.10 (1H, m, H-2*β*), 4.77 (1H, t, *J* = 6.4 Hz, H-3), 7.13 (2H, m, H-5 and H-6), 6.96 (1H, m, H-7), 6.84 (1H, d, *J* = 7.2 Hz, H-8), 4.56 (2H, brs, H-9). ^13^C NMR (*δ*, CDCL_3_, 100 MHz): 47.44 (C-1), 27.89 (C-2), 68.94 (C-3), 162.93 (C-3a), 141.02 (C-4a), 122.50 (C-5), 127.43 (C-6), 123.26 (C-7), 124.83 (C-8), 119.84 (C-8a), 47.44 (C-9). The physical and spectroscopic data were compared with those reported in the literature [21, 22].

**Figure 1:**
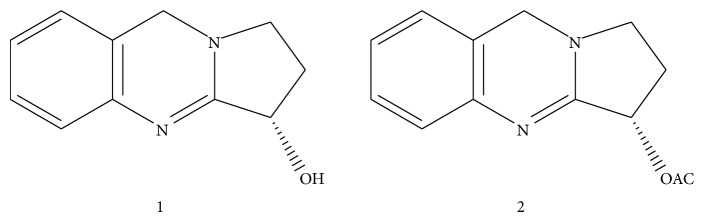
Structure of the compounds: (1) Vasicine and (2) Vasicine acetate.

Vasicine acetate was obtained by acetylation of Vasicine. Molecular formula: C_13_H_14_N_2_O_2_,* M/z* 230. IR: *υ*_max⁡_KBr CM^−1^: 2924, 2858, 1710 (acetate), 1679 (>C=N), 1631, 159, 1467, 1405, 1323, 1234 (acetate), 1185, 1111, 874, 768. ^1^H NMR (*δ*, CDCL_3_, 400 MHz): 3.35 (1H, m, H-1*α*), 3.51 (1H, m, H-1*β*), 2.15 (2H, m, H-2), 4.88 (1H, t, *J* = 6.7 Hz, H-3), 7.19 (2H, m, H-5 and H-6), 7.03 (1H, m, H-7), 6.91 (1H, d, *J* = 7.6 Hz, H-8), 4.66 (1H, m, H-9), 2.06 (3H, m, –OCOCH_3_). ^13^C NMR (*δ*, CDCL_3_, 100 MHz): 49.25 (C-1), 28.53 (C-2), 70.30 (C-3), 163.74 (C-3a), 141 (C-4a), 122.49 (C-5), 128.89 (C-6), 125.15 (C-7), 125.99 (C-8), 117.97 (C-8a), 47.02 (C-9), 179.30 (–OCOCH_3_), 24.00 (–OCOCH_3_). The physical and spectroscopic data were compared with those reported in the literature [15]. The structure of the isolated compound Vasicine and Vasicine acetate (Figure 1).

**Figure 2:**
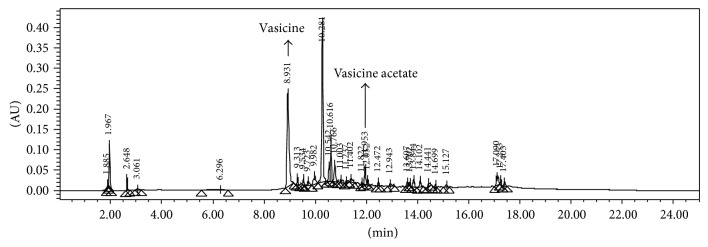
HPLC chromatograms of the ethanol extract of the leaves of* Adhatoda vasica*.

**Figure 3:**
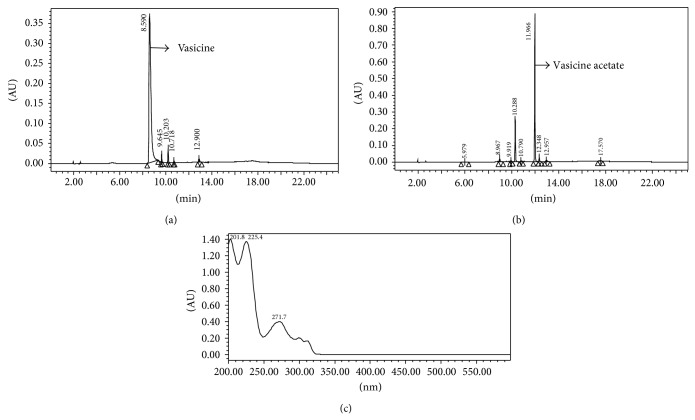
HPLC chromatogram of standard of Vasicine (a), Vasicine acetate (b), and UV spectrum of Vasicine acetate (c).

It was studied for antimicrobial, antioxidant, and anticancer activities. The results of the antimicrobial activity are given in Tables 1 and 2. Vasicine showed antimicrobial activity against* S. aureus, S. epidermidis,* and* K. pneumoniae* (ESBL-3967). Vasicine acetate exhibited activity against bacteria such as 10 mm against* E. aerogenes*, 10 mm against* S. epidermidis,* and 10 mm against* P. aeruginosa*. Vasicine acetate showed minimum inhibitory concentration values against bacteria; they were* M. luteus* (125 *μ*g/mL),* E. aerogenes* (125 *μ*g/mL),* S. epidermidis* (125 *μ*g/mL), and* P. aeruginosa* (125 *μ*g/mL). Karthikeyan et al. [23] reported antimicrobial activity of* A. vasica* leaf extracts against* S. aureus, S. epidermidis, B. subtilis, P. vulgaris,* and* C. albicans*. Ignacimuthu and Shanmugam [15] have reported the antitubercular activity of Vasicine acetate isolated from* A. vasica* leaves. BHT is used as standard of antioxidant. The radical scavenging activity of Vasicine acetate at different concentrations is shown in Table 3. The radical scavenging activity was the maximum at 1000 *μ*g/mL (66.15%). Cytotoxic studies against A549 lung adenocarcinoma cancer cell line showed Vasicine acetate to be the most potent compound (Table 4). Vasicine acetate showed IC_50_ value of 2000 *μ*g/mL. Kulkarni [24] reported that* A. vasica* extract showed good anticancer activity; it showed free radical scavenging activities [25]. Quantification of Vasicine acetate by HPLC-DAD showed its content to be 0.0156%, respectively, on dry weight basis of the leaves. The HPLC chromatogram of the methanol extracts with standards of Vasicine (RT-8.698) and Vasicine acetate (RT-11.903) is given in Figures 2 and 3. Quantification of Vasicine and Vasicine acetate by HPLC-DAD analysis showed their contents to be 0.2293% and 0.0156%, respectively, on dry weight basis of the leaves. Purity of the Vasicine was 98% as shown in Figure 3(a).

## 4. Conclusion

Vasicine acetate was obtained by acetylation of Vasicine recovered from* A. vasica* leaves. Vasicine acetate showed moderate antibacterial activity compared to Vasicine. The radical scavenging activity was maximum at 1000 *μ*g/mL (66.15%). Cytotoxic studies against A549 lung adenocarcinoma cancer cell line revealed that Vasicine acetate had IC_50_ value of 2000 *μ*g/mL. Vasicine acetate could be checked further in drug discovery programme.

## Figures and Tables

**Table 1:** Antimicrobial activity of Vasicine acetate (500 *µ*g/mL) using disc diffusion method (zone of inhibition in mm).

Organism	Vasicine (*µ*g/mL)	Vasicine acetate (*µ*g/mL)	Streptomycin (10 *µ*g/mL)
Gram positive			
*M. luteus *	**—**	9	26
*E. aerogenes *	**—**	10	22
*S. aureus *	**8**	8	14
*S. epidermidis *	**8**	10	14
*B. subtilis *	**—**	9	22
Gram negative			
*S. flexneri *	**—**	8	30
*S. paratyphi-B *	**—**	—	18
*P*. *aeruginosa *	**—**	10	30
*S. typhimurium *	**—**	—	24
Clinical isolates			
*E. coli* (ESBL-3984)	**—**	8	12
*E. coli* (ESBL-3904)	**—**	11	12
*K. pneumoniae* (ESBL-3971)	**—**	—	16
*K. pneumoniae* (ESBL-75799)	**—**	10	16
*K. pneumoniae* (ESBL-3894)	**—**	8	14
*K. pneumoniae* (ESBL-3967)	**8**	—	16
*S. aureus *(MRSA)	**—**	—	—

			Ketoconazole (25 *µ*g/mL)

Fungi			
*C. albicans *	**—**	—	28
*B. cinerea *	**—**	—	22
*T. rubrum *	**—**	—	22
*T. mentagrophytes *	**—**	—	24
*Scopulariopsis *sp.	**—**	—	26
*A. flavus *	**—**	—	28
*M. pachydermatis *	**—**	—	26

(—) no activity; Streptomycin: standard antibacterial agent; Ketoconazole: standard antifungal agent.

**Table 2:** Minimum inhibitory concentration of Vasicine acetate against tested bacteria and fungi.

Organism	Vasicine acetate (*µ*g/mL)	Streptomycin
Gram positive		
*B. subtilis *	250	25
*M. luteus *	125	6.25
*E. aerogenes *	125	25
*S. aureus *	250	6.25
*S. epidermidis *	125	25
Gram negative		
*S. flexneri *	250	6.25
*P*. *aeruginosa *	125	25
Clinical isolates		
*E. coli* (ESBL-3984)	500	25
*E. coli* (ESBL-3904)	500	25
*K. pneumoniae* (ESBL-75799)	250	25
*K. pneumoniae* (ESBL-3894)	500	6.25

		Ketoconazole

Fungi		
*C. albicans *	—	25
*B. cinerea *	—	<12.5
*T. rubrum *	—	<12.5
*T. mentagrophytes *	—	<12.5
*Scopulariopsis *sp.	—	<12.5
*A. flavus *	—	<12.5
*M. pachydermatis *	—	12.5

(—) no activity; Streptomycin: standard antibacterial agent; Ketoconazole: standard antifungal agent.

**Table 3:** Antioxidant activity of Vasicine acetate using DPPH.

Con. *µ*g/mL	DPPH (%)	
	Vasicine acetate	BHT
125	28.27 ± 0.17	60.91 ± 0.31
250	48.31 ± 0.13	71.65 ± 1.07
500	54.53 ± 0.11	80.90 ± 1.93
1000	66.15 ± 0.13	90.14 ± 1.98

Vasicine acetate: isolated compound; BHT: butylated hydroxytoluene.

**Table 4:** Cytotoxic effect of Vasicine acetate against A549 lung adenocarcinoma cancer cell line.

Concentration (*µ*g/mL)	Vasicine acetate	
	%	Mean ± S.D
2000	68.23	0.628 ± 0.00784
1000	44.83	1.091 ± 0.00639
500	33.96	1.308 ± 0.00473
250	26.30	1.457 ± 0.00666
125	23.52	1.512 ± 0.00700
62.5	18.31	1.615 ± 0.00780
